# Spatial Distribution and Chemical Tolerance of *Streptococcus mutans* within Dual-Species Cariogenic Biofilms

**DOI:** 10.1264/jsme2.ME18113

**Published:** 2018-12-08

**Authors:** Yasuhiro Nakanishi, Tatsuya Yamamoto, Nozomu Obana, Masanori Toyofuku, Nobuhiko Nomura, Akihiro Kaneko

**Affiliations:** 1 Graduate School of Medicine, Tokai University 143 Shimokasuya, Isehara, Kanagawa 259–1193 Japan; 2 Faculty of Life and Environmental Sciences, University of Tsukuba 1–1–1 Tennodai, Tsukuba, Ibaraki 305–8572 Japan; 3 Transborder Medical Research Center, Faculty of Medicine, University of Tsukuba 1–1–1 Tennodai, Tsukuba, Ibaraki 305–8572 Japan

**Keywords:** *Streptococcus mutans*, cariogenic biofilm, interspecies interaction, chlorhexidine tolerance, spatial distribution

## Abstract

Bacterial interspecies interactions in the oral cavity influence the structural development of cariogenic biofilms and dental caries. Visualization of the biofilm architecture and bacterial localization within biofilms is essential for understanding bacterial interactions. We herein demonstrated that the spatial localization of *Streptococcus mutans* within dual-species biofilms was altered in a manner that depended on the partner. Furthermore, we found that these biofilms influenced the survival of *S. mutans* against disinfectants. The present results provide information on how *S. mutans* interact with other bacteria in multi-species cariogenic biofilms.

In natural environments, most microbes live in microbial communities called biofilms (17) in which cells are enclosed in self-produced extracellular polymeric substances (EPS), which comprise various substances, including DNA, protein, and polysaccharides (7). Biofilms exhibit increased tolerance to disinfectant chemicals, antibiotics, and host immune systems and are widely recognized as causative agents of infectious diseases and environmental contaminants (11). Based on previous studies on model mono-species biofilms, our current understanding of biofilm physiology is increasing; however, multi-species biofilms are dominant in the natural environment, such as the oral cavity, and interspecies communication shapes the behavior and phenotype of multi-species biofilms (8, 22). Interspecies communication often consequently influences the-architecture of the biofilm community, thereby affecting bacterial spatial distribution (4, 5, 12). Imaging of the spatial distribution of cells in multi-species biofilms is important for fully understanding the role of interspecies interactions. *Streptococcus mutans* is one of the main bacteria causing dental caries, one of the most prevalent oral infectious diseases caused by oral biofilms (9). The human oral cavity contains more than 700 different bacterial species, and each of these bacterial species interacts with at least one other species in the oral cavity (1, 15). Interspecies interactions within cariogenic biofilms containing multiple bacterial species are considered to influence biofilm architecture and properties. A recent study reported that a co-culture with oral bacteria affects gene expression in *S. mutans* (20, 21, 25). However, the effects of these interspecies interactions on the structure of multi-species biofilms and/or spatial distribution of *S. mutans* in cariogenic biofilms remain unclear.

We previously applied a non-invasive real-time imaging system called continuous-optimizing confocal reflection microscopy (COCRM), which allows the visualization of bacteria and attached surfaces using light reflected from a laser (13, 14, 26). We used confocal laser scanning microscopy (CLSM) and COCRM to simultaneously visualize specific oral bacteria and tooth surfaces. *S. mutans* strains that constitutively express fluorescent proteins were constructed for this purpose. We constructed a strain in which *ZsGreen* was expressed under the control of a *ldh* promoter, and was inserted into the SMU_1405c locus of the *S. mutans* genome; this enabled the maintenance of constitutive expression of the fluorescent protein without the use of antibiotics as well as the successful distinction of *S. mutans* from other bacteria, even in thick biofilms on hydroxyapatite (HA) discs (Table S1 and S2). The CLSM image of ZsGreen-tagged *S. mutans* stained with nucleic acid-staining dye SYTO 59 completely merged with the image showing the green fluorescence of cells, indicating that the entire population of *S. mutans* expressed the desired fluorescent protein at the bottom cells in thick biofilms on HA discs (Fig. 1A).

To examine multi-species biofilms, *S. mitis* TU003, *Aggregatibacter actinomycetemcomitans* (*Aa*) ATCC 43718, and *Lactobacillus casei* subsp. *casei* JCM 8129 were selected for the co-inoculation with *S. mutans*. *S. mitis* is a pioneer colonizer of tooth surfaces in the human oral cavity, and its colonization is important for other oral bacteria to adhere to the tooth surface and build dental plaque biofilms (6). *Aa* causes localized aggressive periodontitis (2), and *Aggregatibacter* spp. co-aggregate with *Streptococcus* spp. in human plaques (19). Furthermore, *S. mutans* quorum sensing is activated by *Aa*, suggesting that these bacteria interact with each other in oral biofilms *in vivo* (21). *L. casei* is known to enhance the demineralization of tooth enamel when co-cultured with *S. mutans* (23).

Each overnight culture was diluted OD_600_ of 0.01 in fresh medium and then incubated anaerobically at 37°C for 24 h in polystyrene microtiter plates containing brain heart infusion (BHI) medium (Becton Dickinson, Sparks, MD, USA) on human saliva-coated HA discs (diameter, 7 mm; Clarkson Chromatography Products, South Williamsport, PA, USA) in a 24-well plate (IWAKI non-treated microplate flat bottom, polystyrene) (3). After the incubation, HA discs with biofilms were washed with phosphate-buffered saline (PBS). Biofilms were stained with 0.75 μM propidium iodide (PI; Thermo Fisher Scientific, Waltham, MA, USA) for 30 min. Confocal microscopy images were acquired using the upright confocal laser scanning microscope LSM880 (Carl Zeiss, Oberkochen, Germany) equipped with the W N-Achroplan 40×water-dipping objective lens with a 0.80 numerical aperture (Carl Zeiss). ZsGreen was excited by a two-photon laser (880 nm) and detected with a 499–545 nm band-pass filter, whereas PI was excited by an argon laser (514 nm) and detected with a 590–655 nm band-pass filter. HA disc surfaces were illuminated with an HeNe633 laser (633 nm), and reflected light was collected through a 627–639 nm band-pass filter. A mono-culture of *S. mutans* showed thin biofilm formation under the present experimental conditions, and a few dead cells were observed in biofilm aggregates (Fig. 1A). When *S. mutans* was co-cultured with *S. mitis*, the dual-species biofilm was flat with increased thickness (Fig. 1B). *S. mutans* occupied the bottom layer of the biofilm. PI-stained cells were observed in the upper layer of the biofilm (Fig. 1B). This result indicated that cells in the upper layer of the biofilm were dead cells. Biofilms on HA discs were also subjected to a CFU assay. Biofilms grown in BHI were disrupted by thorough pipetting and incubated anaerobically on BHI agar plates. Since an erythromycin-resistant gene was inserted in the *S. mutans* genome together with *ZsGreen*, erythromycin (10 μg mL^−1^) was added to distinguish *S. mutans* from *S. mitis* and this was followed by a comparison with BHI plates without erythromycin. The plates were incubated at 37°C for 48 h. This assay confirmed that most of the living cells in the biofilm were *S. mutans* (Fig. S1). Thus, the *S. mutans* and *S. mitis* dual-species biofilm showed a layered structure, and live *S. mutans* cells were completely covered by dead cells. When *S. mutans* was co-cultured with *Aa*, negligible effects on biofilm thickness were observed (Fig. 1C). The population of dead cells was slightly greater than that in the *S. mutans* mono-biofilm. Although the co-culture with *S. mutans* showed markedly (10^−5^) lower CFU of *Aa* cells than the monoculture of *Aa*, few *Aa* survived in this dual-species biofilm, suggesting that *S. mutans* co-exists with *Aa* in the dual species biofilm (Fig. S1 and S2). The co-culture of *S. mutans* with *L. casei* showed lower cell density in biofilm aggregates than that of the mono-species biofilm (Fig. 1D). The present results demonstrated that *S. mutans* is the most viable cell in all combinations of dual-species biofilms (Fig. S1), and its localization within biofilms is altered depending on the combination.

We then investigated whether a co-culture of oral bacteria affected the phenotypic properties of *S. mutans*, such as chemical tolerance. We tested the tolerance of mono- and dual-species biofilms to chlorhexidine (CHX), which is frequently used for oral disinfection and is approved by the Food and Drug Administration (24). Briefly, biofilms grown on HA discs were incubated with 0.05% CHX for 5 min, washed with PBS twice, and imaged using CLSM. Confocal images of biofilms and CFU assays showed that most of the cells in *S. mutans* mono-biofilms were dead after the CHX treatment (Fig. 2A, E, and S3). In contrast, cells in the bottom layer of the *S. mutans*-*S. mitis* dual-species biofilm remained alive after the CHX treatment (Fig. 2B). Notably, the co-culture with *S. mitis* significantly increased *S. mutans* viability after the CHX treatment (Fig. 2E and S3). Similarly, significantly increased CHX tolerance was observed in the *S. mutans-Aa* dual-species biofilm (Fig. 2C, E, and S3). However, the co-culture with *L. casei* did not affect the CHX tolerance of *S. mutans* (Fig. 2D, E, and S3). *S. mitis* and *L. casei* mono-species biofilms were highly tolerant to CHX, which was consistent with previous findings (10), suggesting that these biofilms are less permeable to CHX (Fig. S4).

A possible explanation for the increased tolerance of *S. mutans* co-cultured with *S. mitis* is the layered architecture of the dual-species biofilm; dead cells in the upper layer of the biofilm protect *S. mutans* in the bottom layer from chemical disinfectants.

This is consistent with a previous study that demonstrated that peripheral cells in a biofilm protected interior cells from external stresses (18). The dead cells on the upper layer may have been due to bacteriocins and/or H_2_O_2_, which are known to be produced by the *S. mutans* and S. *mitis* groups (*e.g. S. mitis* and *S. sanguinis*), respectively (16). In addition, other mechanisms that increase the CHX tolerance of *S. mutans* in dual-species biofilms may exist because the *S. mutans*-*Aa* biofilm did not show a bilayer structure.

The present results suggest that bacterial spatial distribution within multi-species biofilms differs depending on the combination of bacteria. Moreover, multi-species biofilms exert a strong impact on the tolerance of *S. mutans* to disinfectants. We consider the present study to be useful for advancing research on cariogenic biofilm formation, including that for developing efficient tooth preservation and treatment approaches.

## Supplemental Material



## Figures and Tables

**Fig. 1:**
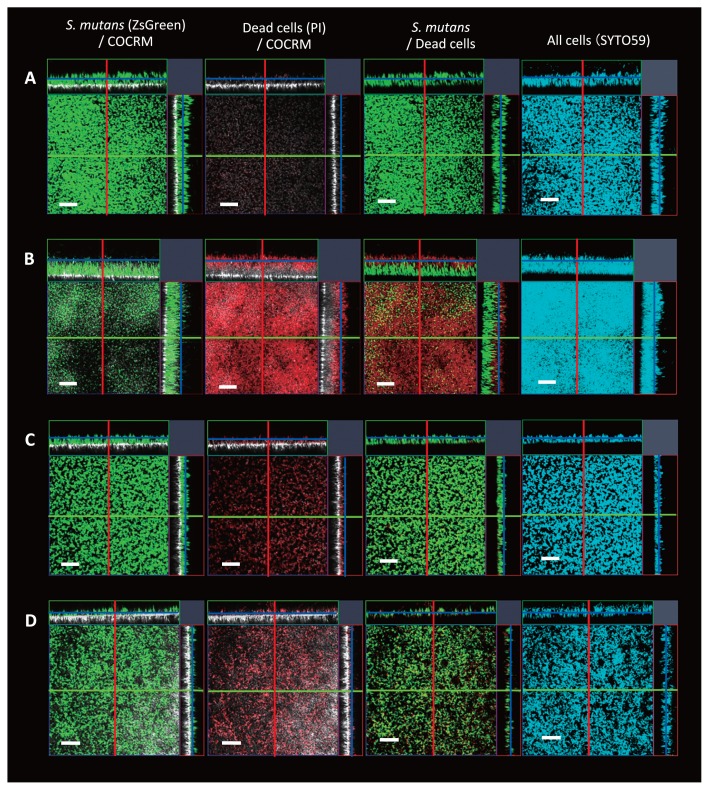
Images showing biofilms and localization of *S. mutans* on hydroxyapatite (HA) discs. (A) Cross-sectional images of a *S. mutans* mono-species biofilm. Blue cells (stained with SYTO59) and green cells (*S. mutans*) are completely merged. (B–D) Dual-species biofilms containing *S. mutans* and (B) *S. mitis*, (C) *Aa*, or (D) *L. casei*. The (A) and (C) layer is 20 μm, the (B) layer is 50 μm, and the (D) layer is 5 μm from the HA disc surface in the vertical lay. Green: *S. mutans* labeled with ZsGreen; Red: dead cells stained with PI; White: HA disc surface; Blue: whole cells with SYTO59. Scale bars indicate 50 μm. The purple lines indicate the positions at which the cross-sections for the X–Y image were taken. Red and green lines indicate the positions at which the X–Z or Y–Z image were taken. Representative images of at least three independent experiments are shown.

**Fig. 2:**
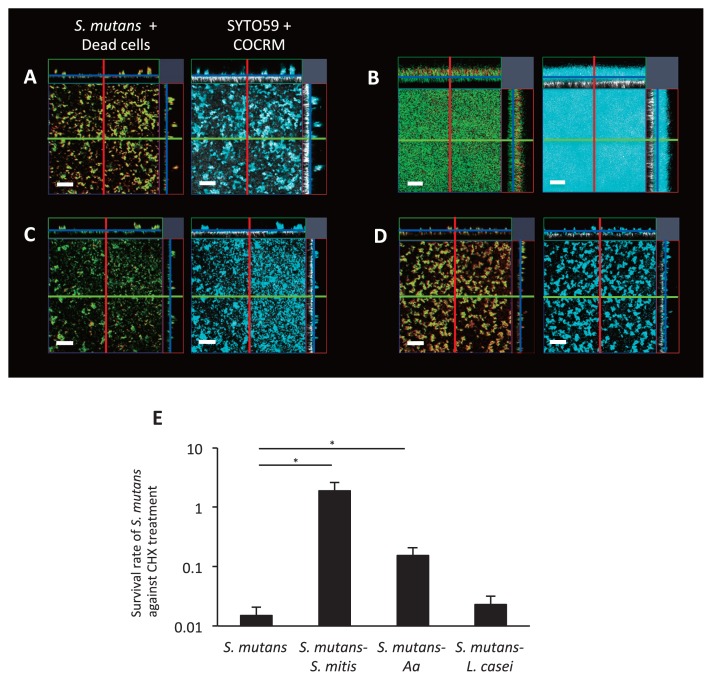
Impact of interspecies interactions in biofilms on the chemical tolerance of *S. mutans*. (A–D) Cross-sectional images of biofilms after the chlorhexidine (CHX) treatment. Representative images of (A) *S. mutans* mono-species biofilms and dual-species biofilms of *S. mutans* with (B) *S. mitis*, (C) *Aa*, or (D) *L. casei*. Green shows *S. mutans* labeled with ZsGreen. Red shows dead cells stained with PI. White shows the HA disc surface taken with COCRM. Blue shows whole cells stained with SYTO59. (E) Survival ratio of *S. mutans* cells on HA disc after the CHX treatment. PBS was used as a control for the CHX treatment and CFU were counted to measure viable cells. The average values with standard errors from at least three independent experiments are shown. The significance of differences (*, *P*<0.05) was evaluated using the Student’s *t*-test.
