# Supplementary material for: Spatial Distribution and Chemical Tolerance of Streptococcus mutans within Dual-Species Cariogenic Biofilms
Source: Microbes Environ. 2018 Dec 8;33(4):455–8. doi: 10.1264/jsme2.ME18113 (PMC6308008; doi:10.1264/jsme2.ME18113)
Supplement: Supplementary file 1 [file 33_455_s1.pdf]

Supplemental Material

Strain construction

Streptococcus mutans YN101: The Smu1405c locus encodes cas9, the deletion of which has no effect on biofilm formation of *S. mutans* (3). To terminate the transcription of *smu1405*, *Bacillus subtilis rpsD* terminator was fused to the region downstream of the 5′ region of *smu1405*. The 5′ region of *smu1405* and the *B. subtilis rpsD* terminator were PCR-amplified using smu_1405 F1/1405_rpsD-term 2 and 1405_rpsD-term 1/rpsD-term_tetR 2 primers sets, respectively. These two PCR products were ligated by overlap PCR using the smu_1405 F2 and rpsD-term_terR 2 primers. The *terR*-Ptet region was PCR-amplified using the rpsD-tetR 1 and Ptet_nonA 2 primers from pWH353 (1). The *nonA-spoVG* terminator region was PCR amplified using the Ptet_nonA 1 and spoVG-term_erm 2 primers from *B. subtilis* TAY3203 (6). PCR products of the *tetR*-P*tet* and *nonA-spoVG* terminator regions were ligated by overlap PCR using the rpsD-term_tetR 1 and spoVG-term_*erm* primers. The erythromycin resistance gene (*erm*) was PCR amplified using the spoVG-term_erm 1 and erm_1405 2 primers from pJIR418 (4). The 3′ region of *smu1405* was PCR amplified using the erm_1405 1 and smu_1405 R1 primers, and the resultant PCR products were ligated by overlap PCR using the spoVG-term_erm 1 and smu_1405 R2 primers. The 5′-*smu1405-rpsD* term, *tetR*-P*tet-nonA-spoVG* term, and 3′-*erm-smu1405* regions were ligated by overlap PCR using the smu_1405 F3 and smu_1405 R3 primers, and the PCR product was introduced into the *smu1405* locus of *S. mutans* UA159 to generate *S. mutans* YN101 by natural competence transformation (2) (Table S1).

S. mutans YN102:

Using the *S. mutans* YN101 strain constructed above, we changed the P_{tet} promoter to the constitutive promoter P_{ldh} and *nonA* to *ZsGgreen*. The *ldh* gene is the most highly expressed constitutive gene (5). The 5′ region of smu1405, Pldh region, and 3′ region of smu1405 were

PCR amplified using the smu_1405 F 1 / rspDterm_Pldh_2, rspDterm_Pldh_1 / Pldh-ZsGreen-2, and ZsGreen-erm-1 / smu_1405 R 3 primers sets, respectively.

The ZsGreen fragments were PCR amplified using the Pldh-ZsGreen-1 and ZsGreen-erm-2 primers from a pZsGreen Vector (Takara Biotechnology Co., Ltd., Shiga, Japan). These four PCR products were ligated by overlap PCR using smu_1405 F 2 / smu_1405 R 2 primers.

The resultant PCR product was introduced into the *smu1405* locus of the *S. mutans* UA159 to generate *S. mutans* YN102 by natural competence transformation (2).

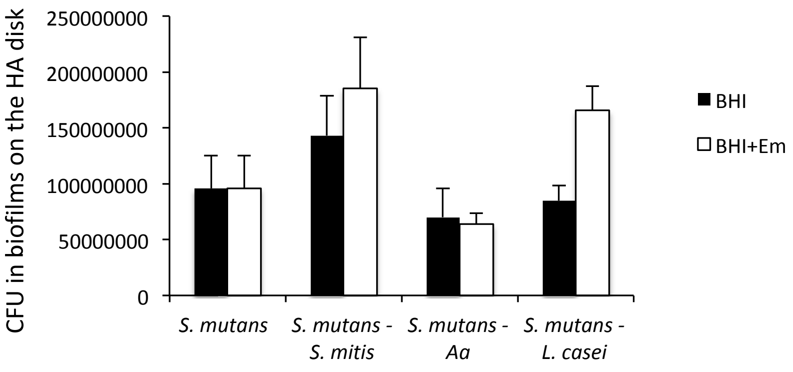

Fig. S1

Viable cell counts within the multi-species biofilms: brain heart infusion (BHI) agar plates were incubated anaerobically, with or without 10 µg/ml erythromycin, at 37°C for 48 h, to measure only the *S. mutans* viable cells (white bars) or to measure the total viable cells (black bars) in the biofilms.

The same data as fig. 2E are used for the CFU value of *S. mutans* (white bars). The average values with standard error from at least three independent experiments are shown.

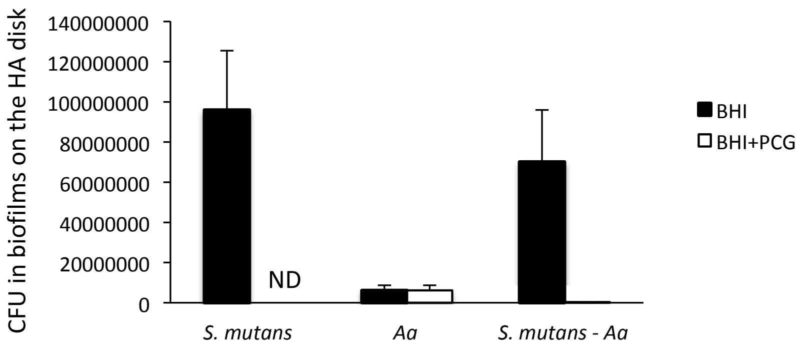

Fig. S2

Viable cell counts. Cells were cultured on brain heart infusion (BHI) agar plates anaerobically, with (white bars) or without (black bars) 0.1 μg/mL penicillin-G, at 37°C for 48 h. To detect only *Aa*, penicillin-G was added to the plates for selection of *Aa*.

Same data as Fig. S1 is used for *S. mutans* and *S. mutans-Aa* biofilms for reference (black bars). The average values with standard error from at least three independent experiments are shown.

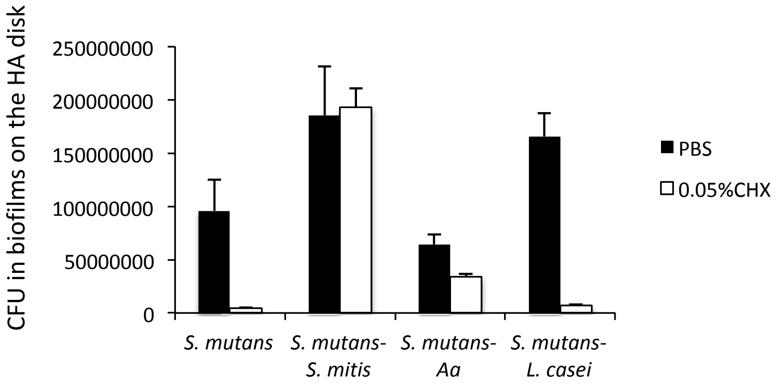

Fig. S3

Viable cell counts of *S. mutans* within mono-the multi-species in biofilms. The *S. mutans* viable cells with (white bars) or without (black bars) the CHX treatment are shown. The survival ratio calculated from this result is shown in Fig. 2E. The average values with standard error from at least three independent experiments are shown.

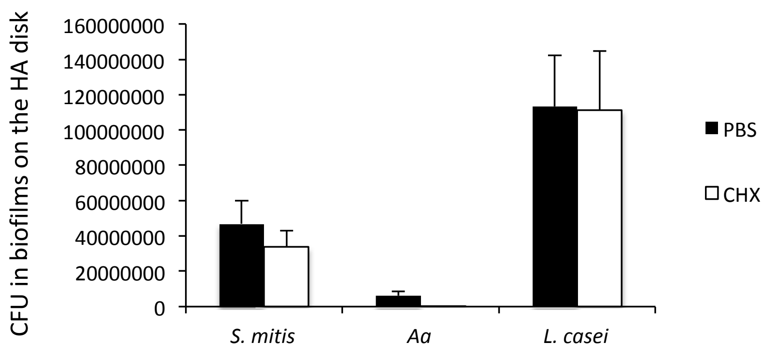

Fig. S4

Streptococcus mitis and *Lactobacillus casei* were resistant to chlorhexidine (CHX) treatment. Mono-species biofilms of *S. mitis, Aggregatibacter actinomycetemcomitans*, and *L. casei* on hydroxyapatite discs were anaerobically grown in brain heart infusion (BHI) medium for 24 h at 37°C. After exposure to 0.05% CHX for 5 min, the biofilms were washed with PBS twice and subjected to the CFU assay. We use same data of the black bars of *Aa* with fig. S2. The average values with standard error from at least three independent experiments are shown.

Table S1. *Streptococcus mutans* strains used in this study

Strain name	Description
S. mutans UA159	WT
S. mutans YN101	*S. mutans* UA159 (*smu1405*::P$_{tet}$-*nonA*, *ermBP*)
S. mutans YN102	*S. mutans* UA159 (*smu1405*::P$_{ldh}$-*ZsGreen*, *ermBP*)

Table S2. Primers used in this study

Primers	Sequence 5′→ 3′
1405_rspD-term 1	gagcgtgaagattttctaagTAATCGTTTTAAAAACCCCT
1405_rpsD-term 2	AGGGGTTTTTAAAACGATTActtagaaaatcttcacgctc
rpsD-term_tetR 1	TCTTGCTGGCCAACACTATgttaactcgacatcttggtta
rpsD-term_tetR 2	taaccaagatgtcgagttaacATAGTGTTGGCCAGCAAGA
Ptet_nonA 1	gagtataattaaaataagctAAACATTCGAAAGGAATGAA
Ptet_nonA 2	TTCATTCCTTTCGAATGTTTagcttattttaattatactc
spoVG-term_erm 1	TTTCAAACTTAGTTGCACTCCAGGAAACAGCTATGACATG
spoVG-term_erm 2	CATGTCATAGCTGTTTCCTGGAGTGCAACTAAGTTTGAAA
erm_1405 1	TCGAAGTGGGCAAGTTGAAAaaagcaacgtacctttgaca
erm_1405 2	tgtcaaaggtacgttgctttTTTCAACTTGCCCACTTCGA
smu_1405 F 1	aaaaccttactctattggacttgatattgg
smu_1405 F 2	tgttgtgacagatgactacaaagttcctgc
smu_1405 F 3	ctgggaaatacagataaaagtcatatcgag
smu_1405 R 1	aaccggatgttctttaagaatttgacttcc
smu_1405 R 2	aacctttcaaacgttgctgtgaatttcgtc
smu_1405 R 3	attttcaggttgatgtcccataattttgac
rspDterm_Pldh_1	CTCTTGCTGGCCAACACTATTTAAAGAGCCCGAGCAACAATAA
rspDterm_Pldh_2	TTATTGTTGCTCGGGCTCTTTAAATAGTGTTGGCCAGCAAGAG
Pldh-ZsGreen-1	AAGGAGATGTTTAGAACATGgctcagtcaaagcacggtct
Pldh-ZsGreen-2	agaccgtgctttgactgagcCATGTTCTAAACATCTCCTT
ZsGreen-erm-1	ccggatctgcattgccctgaAAAATAACCAAAAAGCAAGG
ZsGreen-erm-2	CCTTGCTTTTTGGTTATTTTtcagggcaatgcagatccgg

References

1. Geissendörfer, M., and W. Hillen. 1990. Regulated expression of heterologous genes in *Bacillus subtilis* using the Tn*10* encoded *tet* regulatory elements. Appl. Microbiol. Biotechnol. 33:657-63.
2. Narisawa, N., T. Kawarai, N. Suzuki, Y. Sato, K. Ochiai, M. Ohnishi, H. Watanabe, and H. Senpuku. 2011. Competence-dependent endogenous DNA rearrangement and uptake of extracellular DNA give a natural variant of *Streptococcus mutans* without biofilm formation. J. Bacteriol. 193:5147-5154.
3. Serbanescu, M.A., M. Cordova, K. Krastel, R. Flick, N. Beloglazova, A. Latos, A.F. Yakunin, D.B. Senadheera, and D.G. Cvitkovitch. 2015. Role of the *Streptococcus mutans* CRISPR-Cas systems in immunity and cell physiology. J. Bacteriol. 197:749-761.
4. Sloan, J., T.A. Warner, P.T. Scott, T.L. Bannam, D.I. Berryman, and J.I. Rood. 1992. Construction of a sequenced *Clostridium perfringens-Escherichia coli* shuttle plasmid. Plasmid 27:207-219.
5. Vickerman, M.M., J.M. Mansfield, M. Zhu, K.S. Walters, and J.A. Banas. 2015. Codon-optimized fluorescent mTFP and mCherry for microscopic visualization and genetic counterselection of streptococci and enterococci. J. Microbiol. Methods. 116:15-22.
6. Yamamoto, T., N. Obana, L.M. Yee, K. Asai, N. Nomura, and K. Nakamura. 2014. SP10 infectivity is aborted after bacteriophage SP10 infection induces nonA transcription on the prophage SPβ region of the *Bacillus subtilis* genome. J. Bacteriol. 196:693-706.
